# Electrocatalytic Mn_2_Mo_3_O_8_/MnO-Carbon Nanocomposite Electrodes for Hydrogen Peroxide and Glucose Sensing

**DOI:** 10.3390/molecules31132205

**Published:** 2026-06-23

**Authors:** Foroozan Samimi, Jorge Urraca, Anabel Villalonga, Esther García-Díez, Alfredo Sánchez, Irene Ojeda, Masoud Salavati-Niasari, Reynaldo Villalonga

**Affiliations:** 1 Nanosensors and Nanomachines Group, Department of Analytical Chemistry, Faculty of Chemistry, Complutense University of Madrid, 28040 Madrid, Spain; foroozansamimi@gmail.com (F.S.); jorgeurr@ucm.es (J.U.); anabelvi@ucm.es (A.V.); estgar13@ucm.es (E.G.-D.); alfredos@ucm.es (A.S.); 2 Institute of Nano Science and Nano Technology, University of Kashan, Kashan P.O. Box 87317-51167, Iran; salavati@kashanu.ac.ir; 3 Department of Chemistry in Pharmaceutical Sciences, Analytical Chemistry, Faculty of Pharmacy, Complutense University of Madrid, 28040 Madrid, Spain

**Keywords:** nanocomposite, hydrogen peroxide sensor, glucose biosensor, electrocatalysis, amperometry, food sample

## Abstract

Metal oxide nanomaterials tailored at the nanoscale are opening new avenues for advanced electroanalytical sensing devices with enhanced properties, including improved electrocatalytic activity. In this work, a novel Mn_2_Mo_3_O_8_/MnO-MWCNT nanocomposite was employed to modify a screen-printed carbon electrode, enabling the fabrication of an amperometric sensor for H_2_O_2_ operating at relatively low applied potential due to the catalytic activity of the nanocomposite. Further functionalization of this nanostructured surface with glucose oxidase allowed the construction of an electrochemical glucose biosensor, where the Mn_2_Mo_3_O_8_/MnO-MWCNT material acted as an efficient electrocatalyst for hydrogen peroxide detection. The H_2_O_2_ sensor exhibited a linear response from 0.06 mM to 3.00 mM, with a sensitivity of (2.22 ± 0.02) µA mM^−1^ and a detection limit of 22 µM. The glucose biosensor showed a linear response in the range from 0.10 mM to 18.9 mM glucose, with a sensitivity of (0.345 ± 0.005) µA mM^−1^, and a detection limit of 29 µM. The biosensor displayed excellent selectivity and high stability and was successfully applied to the determination of glucose in lactose-free skimmed milk.

## 1. Introduction

Electrochemical biosensors are increasingly recognized as powerful analytical tools for biomedical diagnostics, food safety, and environmental monitoring due to their high sensitivity, rapid response, and compatibility with miniaturization. Conventional enzyme-based biosensors typically rely on artificial redox mediators to facilitate electron transfer between the enzyme and the electrode surface [1]. Although effective, these mediators often suffer from instability, leaching and complex fabrication procedures [2,3]. To overcome these drawbacks, alternative biosensing strategies have been developed, focusing on the use of electrocatalytically active materials that promote efficient detection of enzymatically generated species without the need for additional redox mediators. These devices exhibit simplified architectures, enhanced stability, and improved reproducibility, making them particularly attractive for practical applications [4].

Recent advances have demonstrated that nanostructured materials can provide the electrocatalytic activity required to construct mediatorless or low overpotential biosensors. Carbon nanomaterials such as graphene oxide, carbon nanotubes (MWCNTs), and activated carbon provide high conductivity, large surface areas, and tunable functional groups that facilitate enzyme immobilization [5,6]. In parallel, transition metal oxides offer intrinsic catalytic activity and versatile redox properties, making them promising candidates to enhance the functionality of hybrid biosensing systems [7,8,9]. Recent reviews have further highlighted the growing interest in nanostructured transition-metal oxides as electrocatalytic materials for electrochemical sensing applications due to their high catalytic activity, tunable composition, and favorable charge-transfer properties [10]. In addition, combining different transition-metal oxides within heterostructured and composite materials has emerged as an effective strategy for enhancing electrocatalytic performance through synergistic interactions and improved charge-transfer kinetics. Such approaches have been successfully applied to the development of high-performance non-enzymatic glucose sensors, highlighting the potential of multicomponent nanostructures as advanced electrochemical sensing platforms [11,12,13,14].

Among these oxides, manganese and molybdenum oxides have attracted particular attention owing to their rich redox chemistry and complementary catalytic roles. Manganese oxides (MnO_x_) provide multiple oxidation states, reversible redox behavior, porous structures, and strong adsorption affinity for ionic species. These characteristics make MnO_x_ attractive not only for energy storage and catalysis [15,16,17] but also for biosensing applications. In oxidase-based biosensors, Mn oxides efficiently catalyze the electrochemical oxidation of hydrogen peroxide, a key product of enzymatic reactions such as the oxidation of glucose catalyzed by glucose oxidase (GOx) [17,18,19]. The electrocatalytic performance of Mn-based systems is highly dependent on their phase composition, Mn^2+^/Mn^3+^/Mn^4+^ redox ratios, crystalline structure, and defect density, which together regulate electron-transfer kinetics and sensor efficiency [20,21].

Molybdenum-based oxides, in turn, offer distinctive structural and electronic features that can further enhance catalytic performance. Oxygen-deficient molybdenum oxides (MoO_3−x_) exhibit both peroxidase- and catalase-like activity, depending on their oxidation state and the density of oxygen vacancies. These vacancies enable reversible Mo^6+^/Mo^5+^ cycling and promote the adsorption and activation of H_2_O_2_ molecules, favoring either their decomposition or reduction. Recent studies have shown that tailoring the Mo valence state allows tuning between oxidizing and antioxidant behavior [22,23].

Hybrid systems that integrate manganese and molybdenum oxides with carbon nanostructures display synergistic properties, combining the conductivity and stability of carbon nanomaterials with the redox versatility and electroactive catalytic surface of the metal oxides [24]. In particular, Mn_2_Mo_3_O_8_/MnO-carbon nanocomposites synthesized via solvothermal methods have been investigated for energy storage applications. Foroozan et al. reported that these composites exhibit disk-like morphologies, high surface areas, and favorable redox characteristics, resulting in excellent performance in lithium-ion and hydrogen storage [25]. These structural and electronic features are also highly attractive for H_2_O_2_ electrochemical sensing.

Several studies have demonstrated the feasibility of Mn-oxide-based nanostructures for glucose biosensing, reporting high sensitivities and rapid response times. However, many of these systems still require relatively high working potentials, higher than 400 mV [17,26,27], which increases the risk of interference from electroactive compounds commonly present in biological or food samples. In parallel, molybdenum-based nanomaterials have also been investigated, showing promising performance in nonenzymatic glucose detection. For instance, α-MoO_3_ nanosheets prepared by atomic layer deposition exhibited remarkable amperometric activity toward H_2_O_2_ at +0.5 V vs. Ag/AgCl, although this relatively high potential poses the same limitation regarding possible interference from coexisting electroactive species [28]. Consequently, the development of materials capable of maintaining efficient electrocatalysis at lower voltages is essential. Designing Mn-based or Mn-Mo hybrid nanocomposites that combine high catalytic activity with low operating potentials therefore remains a major challenge [29].

In this context, the present work describes an amperometric glucose biosensor based on Mn_2_Mo_3_O_8_/MnO nanoparticles integrated with carbon nanostructures, where the nanocomposite acts as an efficient electrocatalyst for the detection of enzymatically generated hydrogen peroxide. The combined Mn and Mo components provide high redox activity toward H_2_O_2_ oxidation, while the carbon matrix ensures efficient electron transport and a large electroactive surface area. Glucose oxidase was selected as the biorecognition element owing to its high specificity for glucose and robustness under immobilization conditions [30]. Accurate glucose determination is not only essential in diabetes diagnostics but also highly relevant in food analysis for quality control and nutritional labeling [31,32].

To the best of our knowledge, this is the first study employing Mn_2_Mo_3_O_8_/MnO nanocomposites as electrocatalytic platforms for glucose biosensing at operating potentials below +0.3 V vs. Ag/AgCl. The biosensor developed here was validated in lactose-free milk, demonstrating its applicability in complex real samples and its potential as a reliable, cost-effective, and stable analytical platform for both food and biomedical monitoring.

## 2. Results and Discussion

**Figure 1:**
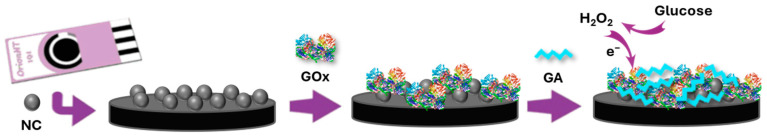
Schematic display of the processes involved in the construction of the amperometric biosensor for glucose.

As is illustrated in Figure 1, the enzyme-based biosensor was fabricated by sequential deposition of Mn_2_Mo_3_O_8_/MnO-MWCNT nanocomposite and glucose oxidase onto the SPCE surface. Among the different carbon-based nanomaterials previously evaluated for this study, the material prepared using multi-walled carbon nanotubes as the carbon source was selected due to its highest specific surface area, improved electronic conductivity, and superior electrochemical performance.

The resulting electrode enables glucose detection through the amperometric oxidation of the hydrogen peroxide produced by the enzymatic reaction and catalyzed by the Mn_2_Mo_3_O_8_/MnO-MWCNT nanocomposite, operating at a potential of +0.25 V vs. Ag/AgCl.

### 2.1. Effect of Biosensor Components on H_2_O_2_ and Glucose Detection

The electrocatalytic contribution of the nanocomposite toward hydrogen peroxide oxidation and its influence on glucose detection were evaluated by cyclic voltammetry (CV) using three modified electrodes: SPCE/GOx, SPCE/NC, and SPCE/NC/GOx. The first configuration contained only glucose oxidase cross-linked with glutaraldehyde, the second incorporated the nanocomposite Mn_2_Mo_3_O_8_/MnO-MWCNT (NC) without enzyme, and the third combined both components, representing the complete biosensor.

**Figure 2:**
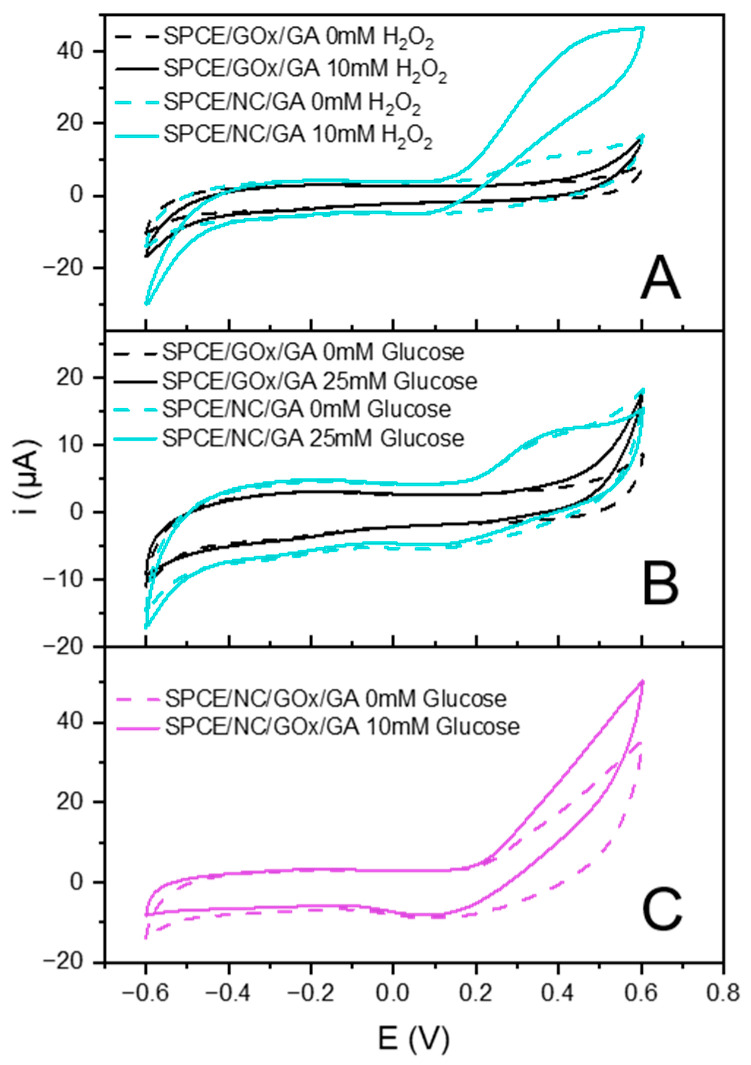
Cyclic voltammograms recorded for (**A**) SPCE/GOx (black) and SPCE/NC (blue) in the absence (dashed line) and presence (solid line) of 10 mM H_2_O_2_, (**B**) SPCE/GOx (black) and SPCE/NC (blue) in the absence (dashed line) and presence (solid line) of 25 mM glucose, and (**C**) SPCE/NC/GOx (violet) in the absence (dashed line) and presence (solid line) of 25 mM glucose. Measurements were performed in 0.1 M sodium phosphate buffer, pH 7.5.

As shown in Figure 2A, the SPCE/GOx electrode exhibited a weak oxidation signal for H_2_O_2_, with the current increase beginning at approximately +0.45 V vs. Ag/AgCl, which reflects the high potential typically required for hydrogen peroxide oxidation on unmodified carbon surfaces. In contrast, the SPCE/NC electrode displayed a well-defined anodic wave at a significantly lower potential of +0.20 V, confirming the electrocatalytic role of the Mn_2_Mo_3_O_8_/MnO-MWCNT nanocomposite, which promotes fast electron transfer and reduces the energy barrier for H_2_O_2_ oxidation.

The voltammetric response of the same electrodes in glucose solution is shown in Figure 2B. The SPCE/GOx electrode exhibited a current response similar to that observed for H_2_O_2_, confirming that the measured signal originated from the oxidation of enzymatically produced hydrogen peroxide. As expected, the SPCE/NC electrode showed no response toward glucose, since it lacked glucose oxidase and therefore could not catalyze its oxidation to produce H_2_O_2_.

When both components were integrated into the same system (SPCE/NC/GOx), a marked anodic current was observed, starting at +0.22 V (Figure 2C), demonstrating the efficient electrocatalytic oxidation of H_2_O_2_ generated during the enzymatic oxidation of glucose. These results confirm that the Mn_2_Mo_3_O_8_/MnO-MWCNT nanocomposite significantly enhances the electrocatalytic oxidation of H_2_O_2_ at low potentials, while the enzyme layer provides high selectivity toward glucose, enabling sensitive and selective detection of this analyte.

As has been previously reported, H_2_O_2_ oxidation proceeds through a multistep mechanism [33]:

H_2_O_2_ + e^−^ → ⋅OH+OH^−^

·OH + e^−^ → OH^−^

2OH^−^ + 2H^+^ → 2H_2_O

4OH^−^ → 2H_2_O + O_2_ + 4e^−^

The final oxidation step is directly associated with the anodic current measured during amperometric detection. The catalytic activity is likely associated with the combined contribution of both phases present in the nanocomposite. Mn_2_Mo_3_O_8_ provides a multivalent Mn redox framework that facilitates electron transfer through Mn^2+^/Mn^3+^/Mn^4+^ transitions, while the Mo–O framework may contribute to the stabilization of the active sites and modulate their electronic environment [34]. MnO may promote H_2_O_2_ adsorption and activation through surface redox processes, facilitating the electrocatalytic oxidation of hydrogen peroxide [35]. Furthermore, the heterojunction formed between MnO and Mn_2_Mo_3_O_8_ may enhance interfacial charge transfer and provide complementary redox-active centers, resulting in improved reaction kinetics and a synergistic enhancement of the electrocatalytic response [36,37]. In this context, the Mn_2_Mo_3_O_8_/MnO-carbon nanocomposite could act as the active electrocatalytic platform, where the high surface area of the nanodiscs and the efficient electron transport provided by the carbon matrix facilitate peroxide adsorption, accelerate interfacial charge transfer, and enhance the final oxidation reaction. Altogether, these findings suggest that Mn_2_Mo_3_O_8_/MnO nanostructures possess excellent catalytic properties, supporting new opportunities for their application in electrochemical biosensors based on H_2_O_2_ generating oxidase enzymes.

### 2.2. Optimization Experiments

The assembly and operating parameters for the biosensor were systematically optimized by comparing the amperometric responses obtained for glucose under different experimental conditions. The slopes of the calibration curves were used to identify the combination of parameters that provided the highest sensitivity. The optimization results are summarized in Table 1 and illustrated in Figure S1 in Supporting Information. All surface modification steps were performed using a deposition volume of 8.0 µL, while the concentrations of the corresponding solutions were varied. The concentration of Mn_2_Mo_3_O_8_/MnO-MWCNT nanocomposite was first optimized (Figure S1A). The maximum sensitivity was obtained at 0.5 mg/mL. However, the small improvement compared to 0.25 mg/mL did not justify doubling the material consumption. Therefore, 0.25 mg/mL was selected as the optimal concentration. Regarding the enzyme loading (Figure S1B), the best response was achieved at a GOx concentration of 0.5 mg/mL. Higher concentrations led to a decrease in sensitivity, which could be associated with enzyme overcrowding and diffusion limitations, which reduce substrate accessibility to the active sites. Similarly, the glutaraldehyde concentration used as the cross-linking agent was optimized (Figure S1C). The highest sensitivity was achieved at 0.5% (*v*/*v*), whereas higher concentrations resulted in lower currents. The decrease in the calibration slope with higher cross-linker concentrations could be attributed to increased rigidity in the enzyme structure, reducing its activity [38]. The influence of the buffer pH on the amperometric response was also evaluated (Figure S1D). The biosensor exhibited its highest current intensity within the pH range of 7.5–8.0, with the maximum signal ratio (presence/absence of glucose) obtained at pH 7.5. This result is consistent with the reported optimum pH for glucose oxidase (GOx) [39,40], confirming that the enzyme maintains its catalytic efficiency and structural stability near physiological pH. This behavior is particularly relevant for practical applications, as it indicates that the biosensor operates efficiently under mild conditions suitable for both biomedical and food analysis. Finally, the effect of the applied potential was investigated (Figure S1E). A working potential of +0.25 V vs. Ag/AgCl was chosen as optimum, as it allows efficient electrocatalysis of H_2_O_2_ while minimizing interference from electroactive species commonly found in complex matrices. This operational potential is lower than that for Mn-based biosensors typically reported in the literature [17,26,41], reflecting the improved electrocatalytic properties of the Mn_2_Mo_3_O_8_/MnO-MWCNT nanocomposite.

### 2.3. Characterization of Analytical Devices

**Figure 3:**
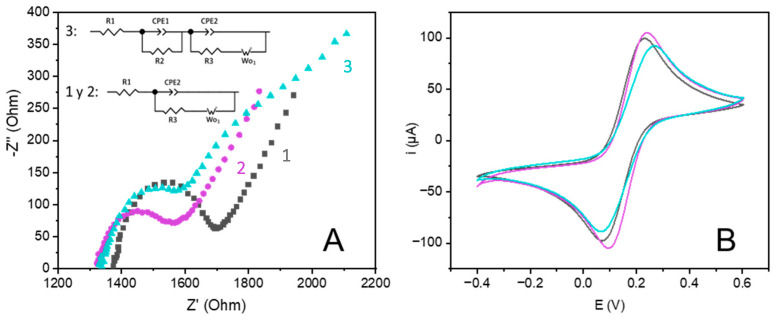
Nyquist plots (**A**) and cyclic voltammograms (**B**) obtained for SPCE (1-black), SPCE/NC (2-violet), and SPCE/NC/GOx (3-blue) in 5 mM Fe(CN)_6_^3−/4−^ in 0.1 M KCl solution. Conditions for EIS (**A**): frequency range of 0.01 to 10^6^ Hz over a frequency range from 0.01 to 10^6^ Hz at the open circuit potential (OCP), using a DC offset of 0.0 V and an AC amplitude of 10 mV. Scan rate for (**B**): 25 mV/s.

The optimized assembly of the SPCE/NC/GOx electrode configuration was further studied by electrochemical impedance spectroscopy (EIS) by using 5 mM [Fe(CN)_6_]^4-/3-^ as redox probe in a 0.1 M KCl solution. The impedance measurements were fitted to a conventional Randles equivalent circuit, and the resulting Nyquist plots are shown in Figure 3A. The modification of the SPCE with the Mn_2_Mo_3_O_8_/MnO-MWCNT nanocomposite resulted in a significant reduction in the electron transfer resistance (Rct), from 261.6 Ω to 160.8 Ω. This decrease indicates that the incorporation of the nanomaterial enhanced the conductivity of the electrode, promoting faster electron transfer at the electrode-electrolyte interface.

Further immobilization of GOx using glutaraldehyde as a cross-linking agent led to an increase in Rct, accompanied by the appearance of a secondary semicircle in the Nyquist plot. This behavior can be explained by the presence of two distinct interfacial processes: the first semicircle at high frequency corresponds to electron transfer at the nanocomposite-modified electrode, while the second semicircle at lower frequency is associated with the enzymatic layer, where the non-conductive enzyme and cross-linker hinder electron diffusion. Accordingly, the impedance spectra can be well modeled using a modified Randles equivalent circuit with two Rct-CPE elements in series, representing the contributions of the nanocomposite and the immobilized enzyme. Despite the increase in resistance, the functionalization with the nanomaterial compensates for this effect, allowing the biosensor to maintain efficient electron-transfer kinetics. Similar behavior has been widely reported in nanostructured enzyme electrodes, where biomolecular layers increase interfacial resistance while conductive nanomaterials facilitate charge transport across the biofilm [42]. These findings underscore the crucial role of the nanocomposite to enhance the electrocatalytic behavior of the biosensor while preserving its sensitivity for glucose detection.

Figure 3B displays the cyclic voltammograms for the SPCE, SPCE/NC, and SPCE/NC/GOx electrodes in 5 mM [Fe(CN)_6_]^3−/4−^ in 0.1 M KCl. All electrodes exhibited a quasi-reversible cyclic voltammetric pattern with well-defined diffusion-limited behavior. The modification with the Mn_2_Mo_3_O_8_/MnO-MWCNT nanocomposite resulted in an increase in the peak current intensities and a reduction in the potential difference between the anodic and cathodic peaks. This enhancement can be attributed to the improved conductivity and electrocatalytic properties of the nanocomposite, which promote more efficient electron transfer. The reduced peak-to-peak separation further confirms a more reversible redox process, emphasizing the positive effect of the nanomaterial on the electrode performance.

However, after coating with GOx and cross-linking with glutaraldehyde, a clear reduction in peak current intensities is observed, along with an increase in peak separation. This change is due to the introduction of non-conductive molecules, which partially block the electron transfer pathways, slowing down electron transfer rates. The increased peak separation suggests a less reversible redox process, probably caused by the added resistance from the enzyme layer, which hinders direct interaction between the redox probe and the electrode surface.

Additionally, cyclic voltammograms were recorded at various scan rates (75, 50, 25, and 12.5 mV/s) for the SPCE, SPCE/NC, and SPCE/NC/GOx electrodes (Figure S2). Table 2 presents the results for the peak-to-peak potential separation (ΔEp) and the ratio of anodic to cathodic peak currents at a scan rate of 25 mV/s. Furthermore, the standard heterogeneous rate constant, k^0^, for the Fe(CN)_6_^3−/4−^ electrochemical reaction, as well as the electroactive surface area, were calculated.

The standard heterogeneous rate constant was determined using the following equations:

(1) $$k^{0}=\Psi \sqrt{\frac{D_{0}\pi \nu F}{RT}{\left(\frac{D_{Red}}{D_{Ox}}\right)}^{\alpha }}$$

(2) $$\Psi =25334\times {\left(n∆E\right)}^{-2.32}$$

where the terms have their usual meanings, and the diffusion coefficients used for the calculations were 6.3 × 10^−6^ cm^2^/s for D_R_ (ferrocyanide) and 7.6 × 10^−6^ cm^2^/s for D_0_ (ferricyanide). The dimensionless kinetic parameter, Ψ, was obtained assuming α = 0.5 and by interpolating the measured ΔEp values from the cyclic voltammograms recorded at different scan rates into the Ψ vs. n·ΔEp data set provided by Nicholson and Perone [43,44] and the potential fit equation (Equation (2)) calculated by Villalonga et al. [45] on the relationship between Ψ and n·ΔE in the range from 98 to 290 mV.

The k^0^ values calculated for the SPCE, SPCE/NC and SPCE/NC/GOx electrodes were (0.9 ± 0.1, 1.2 ± 0.1 and 0.56 ± 0.3) × 10^−3^ cm/s, respectively. These values suggest faster electrode kinetics at the SPCE/NC interface, confirming the positive effect of the Mn_2_Mo_3_O_8_/MnO-MWCNT nanocomposite on the electron transfer rate.

The electroactive surface area was determined by using the Randles–Sevcik equation. A slight increase in the electroactive area was observed upon modification with the Mn_2_Mo_3_O_8_/MnO-MWCNT nanocomposite, from 12.5 mm^2^ (SPCE) to 12.9 mm^2^ (SPCE/NC). However, upon cross-linking-mediated immobilization of glucose oxidase, the electroactive area decreased to 12.1 mm^2^. This behavior reflects the effect of the nanomaterial modification on the enhancement of the electrode surface area and the subsequent reduction due to the presence of non-conductive biomolecules.

The crystal structure of the Mn_2_Mo_3_O_8_/MnO-MWCNT nanocomposite was evaluated by XRD (Figure S3). The obtained diffraction pattern was in good agreement with that previously reported for the Mn_2_Mo_3_O_8_/MnO nanocomposite by Foroozan et al. [19]. The surface of the electrodes was characterized by FE-SEM (Figure S4), showing that the morphology of the nanomaterial was not transformed after enzyme immobilization.

### 2.4. Electrochemical Determination of H_2_O_2_ and Glucose

The SPCE/NC sensor and SPCE/NC/GOx biosensor were evaluated for the amperometric detection of H_2_O_2_ and glucose, respectively. As shown in the amperograms in Figure S5, the steady-state current measured at 250 mV increased progressively with the addition of glucose, indicating efficient electrocatalysis of H_2_O_2_ generated during the enzymatic reaction, which aligns with the mechanism previously proposed.

**Figure 4:**
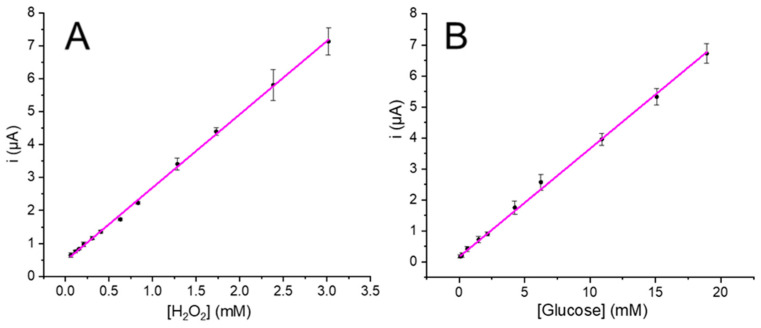
Calibration curve for the H_2_O_2_ sensor (**A**) and glucose biosensor (**B**) in 0.1 M sodium phosphate buffer, pH 7.5, at 0.25 V.

Figure 4A shows the calibration curve for the SPCE/NC-based hydrogen peroxide sensor, demonstrating a linear relationship between the amperometric current response and H_2_O_2_ concentration in the range of 62 µM to 3.0 mM. These data can be fitted to the following equation (r^2^ = 0.9991, *n* = 12):

i (µA) = (2.22 ± 0.02)·[H_2_O_2_, mM] + (0.47 ± 0.03)

In addition, a limit of detection of 22 µM was estimated by using the IUPAC rules [46].

As a control experiment, an SPCE modified only with MWCNTs at the same loading used in the Mn_2_Mo_3_O_8_/MnO-MWCNT nanocomposite was also evaluated toward H_2_O_2_ detection (Figure S6). The MWCNT-modified electrode exhibited a significantly lower sensitivity (42.0 ± 0.7 nA mM^−1^) than the SPCE/NC sensor, confirming that the enhanced electrocatalytic response is mainly associated with the presence of the Mn_2_Mo_3_O_8_/MnO nanocomposite rather than with the carbon material alone.

Figure 4B illustrates the calibration curve for glucose detection using the SPCE/NC/GOx biosensor. This device also exhibited a linear relationship for glucose in the range from 100 µM to 18.9 mM, according to the following equation (r^2^ = 0.998, *n* = 10):

i (µA) = (0.345 ± 0.005)·[Glucose, mM] + (0.16 ± 0.02)

with a limit of detection of 29 µM.

These findings confirm that the incorporation of nanomaterials significantly enhanced the electrocatalytic activity of the sensor for H_2_O_2_ detection, while the inclusion of glucose oxidase allowed efficient glucose detection via the enzymatic generation of hydrogen peroxide. The linear ranges of response, high sensitivities and low limit of detections observed in both systems underscore their potential for precise quantification of these analytes in various applications.

Table 3 summarizes the analytical performance of the SPCE/NC/GOx biosensor developed in this work in comparison with reported electrochemical glucose sensors. The SPCE/NC/GOx biosensor operates at a notably lower applied potential (+0.25 V) than most reported systems (0.4–0.7 V), reducing possible interference from co-existing electroactive species. Despite this lower potential, the sensor exhibits a wide linear range (0.1–18.9 mM) and a low LOD (29 µM), demonstrating comparable or superior analytical performance.

**Figure 5:**
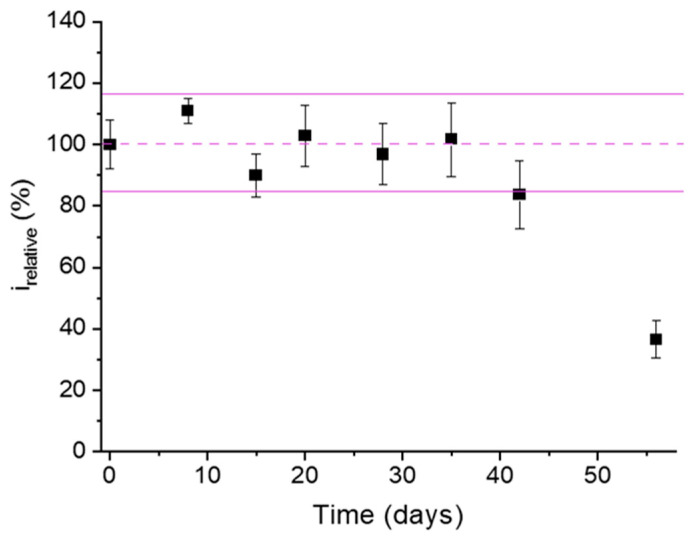
Control chart constructed for the SPCE/NC/GOx biosensor. Each point corresponds to the mean value for three measurements of 11 mM glucose, control limits set by ±2 times the standard deviation of the measurements (*n* = 3) carried out on the first day.

Additional analyses were performed to study the reproducibility of the analytical device for glucose determination by using ten different but identically made sensors. The coefficients of variation obtained for 10.9 mM and 18.9 mM of glucose were 9.2% and 6.3%, respectively, proving the good reproducibility of the SPCE/NC/GOx electrode. The storage stability of this biosensor was also evaluated by storing the modified electrodes in dry conditions at 4 °C. Under these storage conditions, the biosensor showed a stable analytical response over a period of two months, with no significant signal loss for at least 35 days (Figure 5). These results confirm the excellent preservation of both the Mn_2_Mo_3_O_8_/MnO-MWCNT nanocomposite and the immobilized enzyme at low temperature, supporting the robustness of the biosensor for long-term analytical applications.

### 2.5. Selectivity and Cross-Reactivity

**Figure 6:**
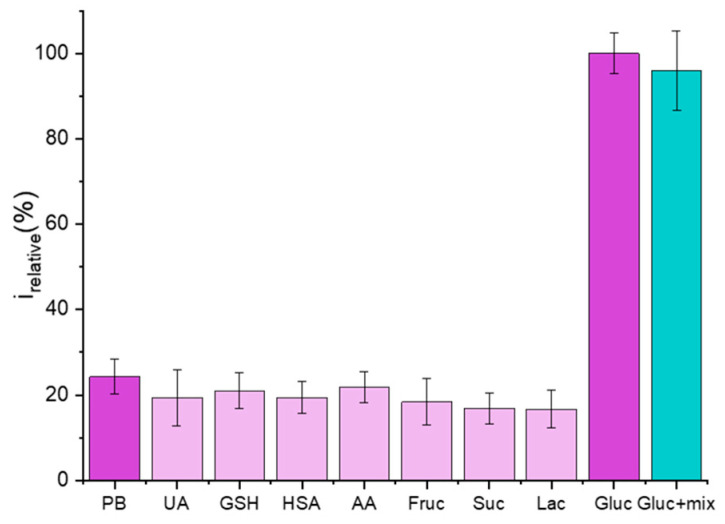
Relative amperometric response of the glucose biosensor in 0.1 M sodium phosphate buffer, pH 7.5, containing glucose at 0 mM (PB) or 2 mM glucose (Gluc and Gluc+mix) in the absence (PB) and the presence of 2 mM uric acid (UA), glutathione reduced (GSH), human serum albumin (HSA), ascorbic acid (AA), fructose (Fruc), Sucrose (Suc) and Lactose (Lac). Bar colors indicate measurements performed in the absence of interferents (dark purple), in the presence of individual interferents (light purple), and in the presence of the interferent mixture (turquoise).

The biosensor demonstrated excellent selectivity against common interfering substances, as shown in Figure 6. Amperometric responses in 0.1 M sodium phosphate buffer, pH 7.5, in the absence of glucose remained constant in the presence of potentially interfering species such as uric acid (UA), reduced glutathione (GSH), human serum albumin (HSA), ascorbic acid (AA), fructose (Fruc), sucrose (Suc) and lactose (Lac). The responses of the glucose sensor in the absence (PB) and presence of glucose (Gluc and Gluc+mix) were not significantly affected by these substances, confirming the selectivity of the sensor. It should be noted that the “Gluc+mix” condition included the simultaneous presence of all tested interferents, each at the same concentration as glucose (2 mM), providing a stringent assessment of the selectivity of the proposed biosensor. Furthermore, at the selected sensing potential, no significant interferences from electroactive compounds such as ascorbic and uric acids were observed.

### 2.6. Determination of Glucose in Lactose-Free Skimmed Milk

**Figure 7:**
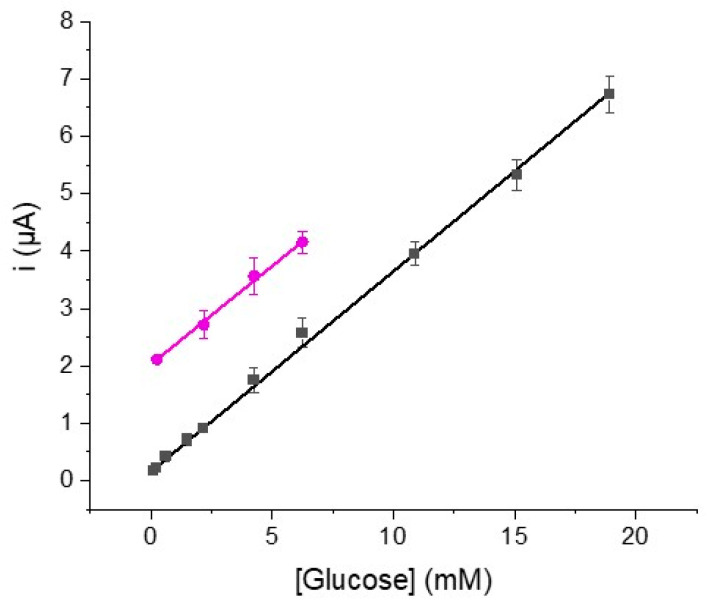
Calibration curve for glucose measured with the SPCE/NC/GOx biosensor in 0.1 M sodium in phosphate buffer, pH 7.5, (black) and in 1:25 dilution of lactose-free skimmed milk (violet) at 0.25 V.

The efficacy of the biosensor for real sample analysis was assessed by analyzing a commercial lactose-free skimmed milk. We first investigated the potential matrix effect. To do that, the slope of the calibration plot obtained by adding known glucose concentrations (ranging from 0.25 to 6.25 mM) to deproteinized lactose-free skimmed milk was statistically compared with that of the calibration curve constructed using glucose standards (Figure 7). The respective slope values were 0.345 ± 0.005 µA mM^−1^ and 0.343 ± 0.008 µA mM^−1^. The t_exp_ value, 0.21, was lower than the tabulated t value (2.179, ν = 12, *p* = 0.05), showing no significant difference at a 95% confidence interval. Based on these findings, glucose concentration in the sample was determined by interpolating the measured current into the calibration plot constructed with standards. The glucose concentration found in the sample was 2.43 ± 0.13 g/dL (*n* = 3), which was not statistically different from the value of 2.4 g/dL indicated in the nutritional information on the product label, thus confirming the accuracy of the developed biosensor.

To further assess the accuracy of the proposed biosensor, recovery values were calculated from the standard addition experiments. Recoveries of 95%, 106% and 100% were obtained for glucose additions of 2.20, 4.25 and 6.25 mM, respectively. These results further confirm the accuracy of the proposed method and indicate that matrix effects do not significantly affect glucose determination under the proposed experimental conditions.

## 3. Materials and Methods

### 3.1. Reagents and Solutions

Mn_2_Mo_3_O_8_/MnO-MWCNTs nanocomposite (NC) was synthesized according to a previously reported solvothermal method [17]. GOx from *Aspergillus niger* (Type VII, ≥100,000 units/g solid), glutaraldehyde solution (25%, grade II in water), glucose, uric acid, ascorbic acid, glutathione reduced, human serum albumin (HSA), and hydrogen peroxide (30%, *w*/*v*) were provided by Sigma-Aldrich (St. Louis, MO, USA). Sodium dihydrogen phosphate and disodium hydrogen phosphate were from Scharlau (Sentmenat, Spain). Lactose-free skimmed milk was purchased in a local supermarket. All other chemicals were of analytical grade. Deionized water was obtained from a Millipore Milli-Q purification system.

### 3.2. Instruments and Electrodes

Electrochemical measurements were performed using screen-printed carbon electrodes (SPCEs) consisting of a carbon working electrode (4.0 mm diameter), a carbon counter electrode, and an Ag/AgCl pseudo-reference electrode (Orion High Technologies S.L., Madrid, Spain). All experiments were conducted with a PalmSens 4 potentiostat (PalmSens BV, Houten, The Netherlands). Field-emission scanning electron microscopy (FE-SEM) images were obtained using a JEOL JSM-7600F microscope (JEOL Ltd., Tokyo, Japan). UV–Vis spectrophotometric analyses were carried out with an Ultrospec^TM^ 8000 Dual Beam spectrophotometer (Biochrom Ltd., Cambridge, UK).

### 3.3. Procedures

#### 3.3.1. Synthesis of Mn_2_Mo_3_O_8_/MnO-MWCNT Nanocomposite

The nanocomposite employed in this work was synthesized according to the solvothermal procedure reported by Foroozan et al. [25], by using multi-walled carbon nanotubes (MWCNT) as the carbon source. Briefly, Na_2_MoO_4_·2H_2_O and Mn(CH_3_CO_2_)_2_·4H_2_O were dissolved in 50 mL of ethylene glycol at a Mo/Mn molar ratio of 3:2. Then, 20 mL of an MWCNT suspension (1 mg/mL) was added, and the mixture was transferred into a Teflon-lined stainless-steel autoclave and maintained at 140 °C for 5 h. The obtained precipitate was washed several times with water and ethanol, dried, and subsequently annealed at 500 °C for 5 h under an argon atmosphere to yield the Mn_2_Mo_3_O_8_/MnO-MWCNT nanocomposite.

#### 3.3.2. Preparation of the Nanocomposite Suspension

An accurately weighed amount of 1.0 mg of Mn_2_Mo_3_O_8_/MnO-MWCNT nanocomposite was dispersed in 1.0 mL of Milli-Q water and sonicated for 5 min in an ultrasonic bath. The resulting dispersion was then diluted with Milli-Q water to obtain a final concentration of 0.25 mg/mL, ensuring uniform deposition and optimal electrocatalytic performance on the electrode surface.

#### 3.3.3. Preparation of Hydrogen Peroxide Sensor and the Glucose Biosensor

The sensing device for hydrogen peroxide was prepared by dropping 8.0 µL of a 0.25 mg/mL of Mn_2_Mo_3_O_8_/MnO-MWCNT suspension on the working electrode surface and drying. To further assemble the glucose biosensor, 8.0 µL of the enzyme solution (0.5 mg/mL in 0.1 M sodium phosphate buffer, pH 7.5) was deposited on the nanostructured working electrode surface and allowed to dry at room temperature. The enzyme was subsequently immobilized through cross-linking with 8.0 µL of 0.5% glutaraldehyde in 0.1 M sodium phosphate buffer, pH 7.5, followed by air-drying under ambient laboratory conditions before electrochemical use, ensuring stable enzyme immobilization on the catalytically active nanocomposite surface.

#### 3.3.4. Amperometric Measurement

Amperometric determinations of hydrogen peroxide and glucose were carried out at a constant potential of +0.25 V vs. Ag/AgCl in 45 µL of 0.1 M sodium phosphate buffer, pH 7.5. The analyte was successively added to the electrochemical cell, and the steady-state current was recorded after 100 s.

#### 3.3.5. Milk Sample Treatment

Lactose-free skimmed milk was sonicated for 10 min and deproteinized with 20% trichloroacetic acid. After 15 min of protein precipitation, the mixture was centrifuged at 9000 rpm for 2 min, and the supernatant was collected. A 1:25 dilution of the clear supernatant was then prepared using 0.1 M sodium phosphate buffer, pH 7.5, prior to electrochemical analysis using the electrocatalytic glucose biosensor.

## 4. Conclusions

This work demonstrates that screen-printed carbon electrodes modified with Mn_2_Mo_3_O_8_/MnO-MWCNT nanocomposite provide efficient and stable electrocatalytic activity toward H_2_O_2_ oxidation at low potential, enabling sensitive amperometric detection. By integrating this nanocomposite with glucose oxidase, a simple, low-cost, and rapid glucose biosensor was developed, operating effectively at +0.25 V vs. Ag/AgCl.

The resulting device exhibited enhanced electron-transfer kinetics, low operating potential, and reliable analytical performance, enabling sensitive glucose detection through enzymatic generation and subsequent electrocatalytic oxidation of H_2_O_2_. Validation in commercial lactose-free milk confirmed its applicability in real samples without requiring complex pretreatment.

In summary, the Mn_2_Mo_3_O_8_/MnO-MWCNT nanocomposite acts as a bifunctional electrocatalyst, combining redox activity and conductivity to support efficient oxidase-based biosensing. These findings highlight its potential for the development of versatile, low-cost, and stable electrochemical sensors, suitable for biomedical and food-analysis applications.

## Figures and Tables

**Table 1:** Optimized experimental conditions for the assembling and use of the SPCE/NC/GOx-based electroanalytical biosensor for glucose.

Parameter	Units	Range	Optimal Value
NC	mg/mL	0.00–0.50	0.25
GOx	mg/mL	0.25–2.00	0.50
GA	%	0.25–2.00	0.50
pH	-	5.00–9.00	7.50
Working potential	V	0.15–0.25	0.25

**Table 2:** Parameters estimated for SPCE, SPCE/NC, and SPCE/NC/GOx electrodes measured by cyclic voltammetry at different scan rates in 5 mM [Fe(CN)_6_]^4−/3−^ in 0.1 M KCl solution.

	ΔE (mV)	I_pa_/I_pc_	$k^{0}$ 10^−3^ (cm/s)	A (mm^2^)
SPCE	155	1.027	0.9 ± 0.1	12.5 ± 0.5
SPCE/NC	145	1.017	1.2 ± 0.1	12.9 ± 0.5
SPCE/NC/GOx	195	1.040	0.56 ± 0.03	12.1 ± 0.3

**Table 3:** Analytical performance comparison of the SPCE/NC/GOx biosensor with recently reported electrochemical glucose sensors.

Electrode	E_app_ (V)	Reference	Range (mM)	LOD (µM)	Sample	Ref.
Pt/PPy-GOx/PPy-Cl	+0.70	Ag/AgCl	0.5–24	26.9	Fruit juices	[47]
Pt/GOx/AuNPs/BSA/Fe_3_O_2_	+0.40	Ag/AgCl	0.25–7.0	3.54	-	[48]
GOx/Naf/MnO_2_/GNR/SPCE	+0.50	Ag/AgCl	0.1–1.4	50	Honey samples	[26]
GOx/MnO_2_/ITO	+0.45	Ag/AgCl	0.1–3.0	-	-	[17]
GCE/MnO_2_-CNFs/GOx/CS	+0.60	SCE	0.08–4.6	15	Spiked human urine	[49]
GCE/MnO_2_/Naf/GOx	+0.70	Ag/AgCl	0.2–3.8	25.56	Spiked human urine	[41]
CPE/GOx-SiO_2_/Lig/Fc	+0.60	Ag/AgCl	0.5–9	145	Liquid Glucose 5% Glucose solution	[50]
GCE/MnO_2_/GOx/Naf	+0.65	SCE	0.005–2	1.8	Human Serum Peach juice	[27]
SPCE/NC/GOx/	+0.25	Ag/AgCl	0.1–18.9	29	Lactose-free milk	This work

AuNPs: Gold nanoparticles; CNFs: carbon nanofibers; CS: chitosan; Fc: Ferrocene; GNR: Graphene nanoribbons; ITO: indium tin oxide coated glass; Lig: Lignin; Naf: Nafion; PPy: polypyrrole; SCE: saturated calomel electrode.

## Data Availability

Data are contained within the article and Supplementary Materials.

## Supplementary Materials

The following supporting information can be downloaded at https://www.mdpi.com/article/10.3390/molecules31132205/s1, Figure S1. Optimization of experimental parameters for the glucose biosensor assembly and amperometric response toward glucose. Figure S2. Cyclic voltammograms of SPCE, SPCE/NC and SPCE/NC/GOx/GA at different scan rates in a 5 mM Fe(CN)_6_^3−/4−^ solution. Figure S3. XRD pattern for synthesized Mn_2_Mo_3_O_8_/MnO-MWCNTs. Figure S4. Representative SEM images of SPCE, SPCE/NC and SPCE/NC/GOx/GA. Figure S5. Amperometric responses of the hydrogen peroxide sensor and glucose biosensor at different analyte concentrations. Figure S6: Calibration curve for the H_2_O_2_ obtained with SPCE-MWCNT in 0.1 M sodium phosphate buffer, pH 7.5, at 0.25 V.
