# Supplementary material for: Electrocatalytic Mn2Mo3O8/MnO-Carbon Nanocomposite Electrodes for Hydrogen Peroxide and Glucose Sensing
Source: Molecules. 2026 Jun 23;31(13):2205. doi: 10.3390/molecules31132205 (PMC13362840; doi:10.3390/molecules31132205)
Supplement: Supplementary file 1 [file molecules-31-02205-s001.zip › molecules-4345320-supplementary.pdf]

Electrocatalytic $Mn_2Mo_3O_8$/MnO–carbon nanocomposite electrodes for hydrogen peroxide and glucose sensing

Foroozan Samimi[1,2], Jorge Urraca[1], Anabel Villalonga[1], Esther García-Díez[1], Alfredo Sánchez[1], Irene Ojeda[1,3*], Masoud Salavati-Niasari[2], Reynaldo Villalonga[1*]

[1]Nanosensors and Nanomachines Group, Department of Analytical Chemistry, Faculty of Chemistry, Complutense University of Madrid, 28040 Madrid, Spain.

[2]Institute of Nano Science and Nano Technology, University of Kashan, Kashan, P. O., Iran.

[3]Department of Chemistry in Pharmaceutical Sciences, Analytical Chemistry, Faculty of Pharmacy, Complutense University of Madrid, 28040 Madrid, Spain

* Corresponding authors: rvillalonga@quim.ucm.es; ireojeda@ucm.es

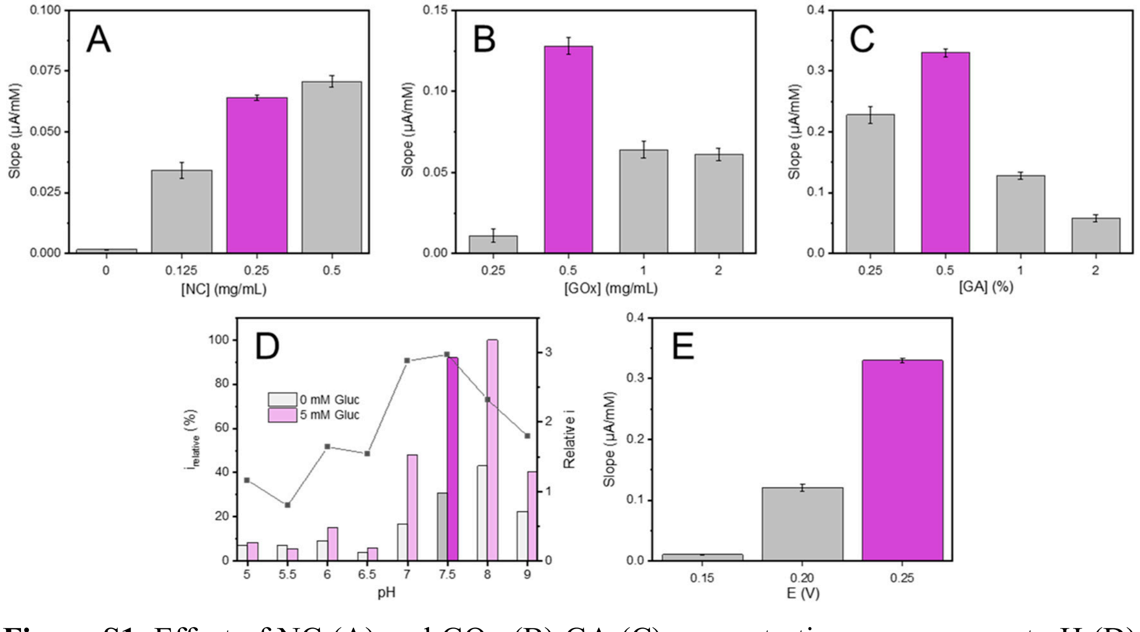

Figure S1. Effect of NC (A) and GOx (B) GA (C) concentration, measurement pH (D) and applied potential (E) on the amperometric response of the sensor toward glucose. The dark violet color corresponds to the selected optimal value used in subsequent measurements.

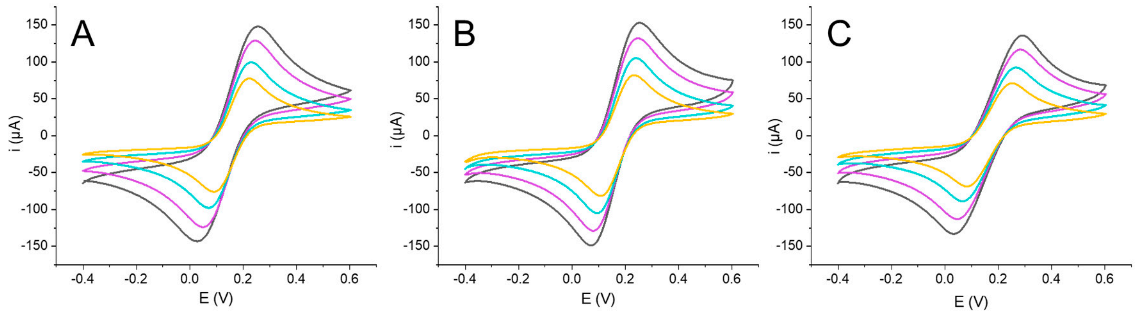

Figure S2. Cyclic voltammograms for SPCE (A) SPCE/NC (B) and SPCE/NC/GOx/GA (C) in 5 mM $Fe(CN)_6^{3-/4-}$ in 0.1 M KCl at different potential scan rates (75 mV/s (black); 50 mV/s (violet); 25 mV/s (blue) and 12.5 mV/s (yellow)).

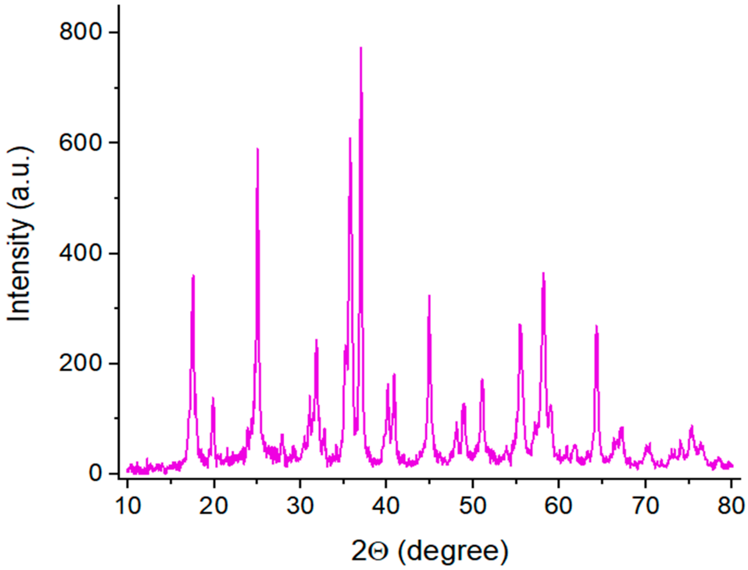

Figure S3. XRD pattern for synthesized $Mn_2Mo_3O_8$/MnO-MWCNTs

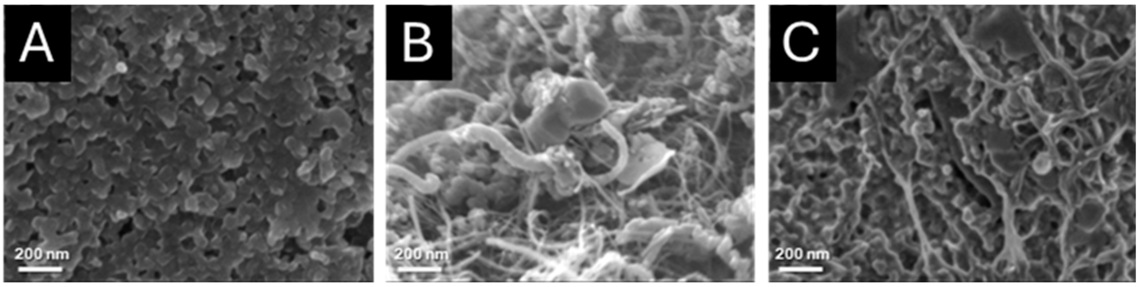

Figure S4. Representative SEM images of SPCE (A), SPCE/NC (B) and SPCE/NC/GOx/GA (C).

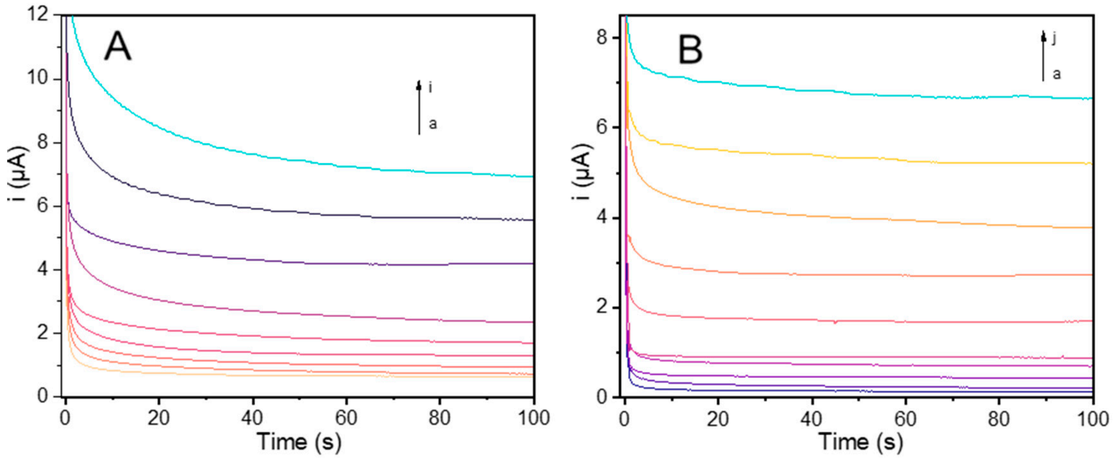

Figure S5. Amperometric recording at 0.25 V with the hydrogen peroxide sensor in 0.1 M sodium phosphate buffer, pH 7.5 at different concentrations of H_2O_2 a) 62 µM, b) 115 µM, c) 210 µM, d) 400 µM, e) 630 µM, f) 830 µM, g) 1.30 mM, h) 1.75 mM and i) 3.00 mM (A) and the glucose biosensor in 0.1 M sodium phosphate buffer, pH 7.5 at different concentrations of glucose: a) 100 µM, b) 215 µM, c) 625 µM, d) 1.50 mM, e) 2.20 mM, f) 4.25 mM, g) 6.25 mM, h) 10.90 mM, i) 15.09 mM and j) 19.00 mM (B).

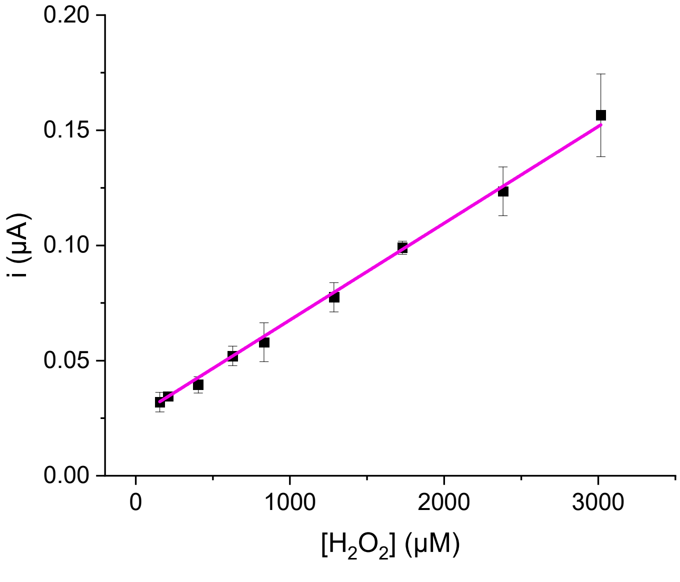

Figure S6. Calibration curve for the H_2O_2 obtained with SPCE-MWCNT in 0.1 M sodium phosphate buffer, pH 7.5, at 0.25 V.
